# Baicalin enhances proliferation and reduces inflammatory-oxidative stress effect in H_2_O_2_-induced granulosa cells apoptosis via USP48 protein regulation

**DOI:** 10.1186/s12906-024-04346-z

**Published:** 2024-01-20

**Authors:** Jun Chen, Chuhua Lin, Xiurong Huang, Wei Bian

**Affiliations:** 1 Department of Traditional Chinese Medicine, Shenzhen People’s Hospital (The Second Clinical Medical College of Jinan University; The First Affiliated Hospital of Southern University of Science and Technology), No. 1017, Dongmen North Road, Luohu District, Shenzhen, 518020 China; 2 Department of Rehabilitation Medicine, Shenzhen People’s Hospital (The Second Clinical Medical College of Jinan University; The First Affiliated Hospital of Southern University of Science and Technology), Shenzhen, 518020 China

**Keywords:** USP48, Baicalin, Oxidative stress, Inflammation, Ovarian granulosa cell

## Abstract

**Background:**

Oxidative stress and inflammation can lead to apoptosis of ovarian granulosa cells (GCs), resulting in ovulation disorders and infertility. Baicalin (BAI) promotes cell proliferation and reduces inflammation and oxidative stress. However, the mechanisms by which BAI treatment affects oxidative stress and inflammation in GCs remain incompletely understood.

**Methods:**

KGN cells were treated with hydrogen peroxide (H_2_O_2_) to analyze the effect of oxidative stress on GCs in vitro. Subsequently, H_2_O_2_-stimulated KGN cells were treated with BAI. The levels of GSH-Px, CAT, and SOD were measured using an activity assay kit. The levels of MDA, IL-1β, IL-6, IL-8, and TNF-α were measured by ELISA. Proliferation, apoptosis, and mRNA and protein levels were measured using the CCK8, flow cytometry, qRT-PCR, and western blotting.

**Results:**

H_2_O_2_ treatment inhibited KGN cell proliferation and promoted apoptosis, accompanied by increased oxidative stress and inflammation. BAI promoted proliferation, inhibited apoptosis, and reduced oxidative stress and inflammation in H_2_O_2_-stimulated KGN cells. BAI treatment promoted USP48 protein expression, and USP48 knockdown abrogated the protective effects of BAI, indicating that USP48 is a downstream mediator of BAI.

**Conclusion:**

BAI treatment enhanced cell proliferation and ameliorated oxidative stress and inflammation by enhancing USP48 protein expression. BAI, which is used clinically and as a dietary supplement, may alleviate oxidative stress-induced GC injury and ovarian disorders.

**Supplementary Information:**

The online version contains supplementary material available at 10.1186/s12906-024-04346-z.

## Background

 Follicles are the structural and functional units of the ovary and include oocytes and granulosa cells (GCs). GCs synthesize various hormones and growth factors, including estrogen and progesterone, to regulate the growth, differentiation, and maturation of oocytes, thereby regulating follicular development [1]. GC apoptosis leads to oocyte dysfunction and reduction in ovarian reserve [2, 3]. For example, GC apoptosis and dysfunction can induce follicular dysplasia in childbearing women with polycystic ovarian syndrome (PCOS) [4]. Therefore, GSs are essential for the initiation and development of follicles, and their dysfunction or apoptosis is a key factor in follicular atresia. Studying apoptosis and GC dysfunction is crucial to improve ovarian function and oocyte development. Abnormal oxidative stress is an important cause of abnormal function and apoptosis in GCs. Oxidative stress in the ovarian microenvironment can cause GC apoptosis and dysfunction, thereby affecting normal oocyte development and ovarian functions [5, 6]. Oxidative stress and chronic inflammation have been observed in patients with PCOS owing to the influence of obesity and hyperinsulinemia, which induce apoptosis and dysfunction of GCs, leading to a decline in oocyte quality or capacity. Oxidative stress in GCs can lead to reproductive system disorders in women such as PCOS and premature ovarian failure [7]. Therefore, reducing inflammation and oxidative stress in GCs may improve oocyte quality and function.

Baicalin (BAI) is a flavonoid extracted from the radix of *Scutellaria baicalensis* [8]. In many diseases, BAI enhances cell proliferation while dampening inflammation and oxidative stress. BIA can effectively alleviate infection-associated lung injury by inhibiting inflammation, oxidative stress, immune responses, and cell apoptotic pathways [9]. BAI alleviates central nervous system disorders via its anti-neuroinflammatory and anti-neuronal apoptotic properties and mitigates metabolic disorders via anti-inflammatory and antioxidant mechanisms [10]. It also regulates the production of the gut microbiota and short-chain fatty acids, thereby relieving the symptoms of gastrointestinal disorders by improving chronic inflammation, immune imbalance, lipid metabolism disorders, cell apoptosis, and oxidative stress [11]. Hence, BAI exhibits potent anti-inflammatory and antioxidant properties. In addition, BAI treatment can enhance the secretion of estradiol and progesterone and the activity of GCs and improve the estrous cycle and oocyte quality via the mTOR pathway, thereby promoting the improvement of ovarian function [12]. BAI improves hormone imbalance, prolongs the estrous cycle, insulin resistance, and inflammation in mice with PCOS by activating the AMPK pathway and improving ovarian histological changes and follicular development [13]. BAI treatment in a rat model of hyperandrogenic PCOS significantly reversed elevated serum androgen levels and ovarian abnormalities and restored the estrous cycle via the GATA1/HSD3B2 axis [14]. BAI treatment reduces inflammation and testosterone concentrations and improves hormone secretion and follicular development in rats with PCOS via the miR-874-3p/FOXO3 and miR-144/FOXO1 axes [15]. However, the mechanisms by which BAI treatment affects oxidative stress and inflammation in GC remain incompletely understood.

KGN is a human ovarian granulosa-like tumor cell line. KGN cell exhibits a steroidogenesis pattern similar and the physiological regulation of apoptosis similar to that observed in normal human GCs, therefore, this cell line is considered a useful model for studying the steroidogenesis, growth, and apoptotic regulation of human GCs in vitro [16]. In addition, exposure to H_2_O_2_ increases the inflammatory factors, free radicals, ROS levels, and oxidative stress in GCs, thereby promoting GC apoptosis [17, 18]. Hence, H_2_O_2_-stimulated KGN cells were used to analyze the effects of oxidative stress on GCs in vitro. Proliferation, apoptosis, inflammation, and oxidative stress were measured after BAI treatment of H_2_O_2_-stimulated KGN cells. In addition, potential target genes of BAI in H_2_O_2_-stimulated KGN cells were screened and verified. Here, using Gene Expression Omnibus (GEO) data, a differentially expressed gene *UPS48* related to polycystic ovary syndrome (PCOS) was discovered. USP48 is a deubiquitinating enzyme with a catalytic core ubiquitin C-terminal hydrolase domain located at the N-terminus [19]. USP48 possesses an ubiquitin-like (UBL) domain at its C-terminal end, which adopts a three-dimensional structure similar to ubiquitin and provides a regulatory mechanism for its catalytic activity [19]. USP48 is involved in various cellular processes, including DNA repair, cell cycle regulation, immune responses, and tumorigenesis [20–23]. These results revealed the key regulatory gene, *USP48*, by which BAI affects apoptosis in GCs.

## Materials and methods

### Compound

BAI (C_21_H_18_O_11_; molecular weight: 446.37 g/mol; purity: HPLC ≥ 98%) was purchased from Taian Biotechnology Co., Ltd. (Shanxi, China) and dissolved in DMSO.

### Cell culture, transfection, and treatment

KGN cells (AnWei-Sci, Shanghai, China) were cultured in DMEM/HamF12 + 10% FBS at 37 ℃ in an atmosphere containing 5% CO_2_. After three passages, KGN cells were treated with different concentrations of H_2_O_2_ for 24 h to mimic the oxidative stress microenvironment (H_2_O_2_ group). H_2_O_2_ concentrations ranging from 100 to 500 µmol/L significantly inhibit the proliferation of GCs [17, 18]. Therefore, in this study, we selected a concentration range of 50–800 µmol/L of H_2_O_2_ to analyze its effects on KGN cell proliferation. The untreated KGN cells served as controls (control group). Subsequently, the H_2_O_2_-stimulated KGN cells were treated with different concentrations of BAI (0–80 µmol/L, H_2_O_2_ + BAI group) 24 h to analyze the effects of BAI. In the BAI + 0 µmol/L BAI group, a concentration of 0 µmol/L signified the introduction of solely dimethyl sulfoxide (DMSO). To identify the impact of USP48 on BAI-treated cells, 20 µM siRNA negative control (siNC group, RIBOBIO, Guangzhou, China) and 20 µM siUSP48 (siUSP48 group, RIBOBIO) were transfected into KGN cells, which were then treated with H_2_O_2_ and 20 µmol/L of BAI. Untransfected KGN cells served as blank controls and were treated with H_2_O_2_ and 20 µmol/L BAI (Blank group). The siUSP48 sequences were as follows: siUSP48-1, 5ʹ-GCATATTTGGTTAGGAGAA-3ʹ; and siUSP48-2, 5ʹ-GGTGAATGGTATAAGTTTA-3ʹ.

### CCK8 and flow cytometry assays

KGN cell proliferation was analyzed using a Cell Counting Kit-8 (CCK-8; Dojindo, Japan) after H_2_O_2_ and/or treatment with 20 µmol/L BAI. Briefly, KGN (100 µl, 1 × 10^4^ cells) cells were inoculated in a 96-well plate and cultured at 37 ℃ in a conditioned incubator containing 5% CO_2_. After treatment for 24 h, 10 µL of CCK-8 was added to every well, and the culture plate was incubated in a multiscan MK3 incubator (Thermo Fisher Scientific, Waltham, MA, USA) for 4 h. Absorbance was measured at 450 nm using a microplate reader. KGN cell apoptosis was analyzed using an Annexin V/PI apoptosis kit (BD, Franklin Lakes, NJ, USA). In brief, KGN was washed twice with cold PBS and then resuspended cells in 1X Binding Buffer at a concentration of 1 × 10^6^ cells/ml. Then, 100 µl of the solution (1 × 10^5^ cells) was transferred to a 5 ml culture tube and added 5 µl of FITC Annexin V and 5 µl PI. KGN was gently vortexed and incubate for 15 min at 25 °C in the dark. Finally, 400 µl of 1X Binding Buffer was added to each tube and a FACSCalibur flow cytometer (BD). Reactive oxygen species (ROS) level in KGN cells was analyzed using a DCFH-DA ROS Assay Kit (Beyotime, Shanghai, China) and FACSCalibur flow cytometer (BD). All reactions were performed in triplicate.

### ELISA assay

The activities of glutathione peroxidase (GSH-Px), catalase (CAT), and superoxide dismutase (SOD) were measured using GSH-Px, CAT, and SOD activity assay kits (Catalog No.: E-BC-K096-M, E-BC-K031-M, E-BC-K020-M; Elabscience, Wuhan, China), respectively. Malondialdehyde (MDA), interleukin (IL)-1β, IL-6, IL-8, and tumor necrosis factor-α (TNF-α) levels were measured using MDA, IL-1β, IL-6, IL-8, and TNF-α ELISA kits (Catalog No.: E-EL-0060c, E-EL-H0149c, E-EL-H6156, E-EL-H6008, E-EL-H0109c, Elabscience). After cell culture at 24 h, the culture supernatant was collected and centrifuged at 1000 × *g* for 20 min to remove impurities and cellular debris. The supernatant was collected for further analysis. For the detection, 50 µl of the supernatant was taken and analyzed. Following completion of the experiment, the optical density (OD) of each well was measured using a Multiscan MK3 reader (Thermo Fisher Scientific). All reactions were repeated thrice.

### qRT-PCR assay

Total RNA was extracted from KGN cells using the Trizol Reagent (Beyotime). The mRNAs were then reverse-transcribed using EasyScript First-Strand cDNA Synthesis SuperMix (TransGen, Beijing, China) at 30˚C for 10 min, 42˚C for 30 min, and then 85˚C for 5 s. The expression levels of SNX29, MDM2, and USP48 were measured using the SYBR Green qPCR SuperMix (Vazyme, Nanjing, China) on an ABI PRISM 7500 Sequence Detection System (Roche, Basel, Switzerland). The reaction system (20 µl) was prepared according to the instructions provided with the reagent kit. The cycling conditions were as follows: Initial denaturation at 95˚C for 5 min, followed by 40 cycles at 95˚C for 15 s and 60˚C for 32 s. 18 S RNA was used for normalization of the expression of SNX29, MDM2, and USP48. All reactions were repeated thrice. The primers used for qRT-PCR are listed in Table 1.

**Table 1:** qRT-PCR primers

Gene	Primers	Size
SNX29-F1	GGGATGGGGGTAGAGCTAAT	122 bp
SNX29-R1	AAGTCGGTGTCAGGTGAGTA	
MDM2-F1	AGCCTCCAATGAGAGCAACT	144 bp
MDM2-R1	TGTCCCAGCTACCTCCCTTA	
USP48-F1	TCGTGGTGAGAAAGCACTTC	174 bp
USP48-R1	ACAGATTCAGGAATGACGCC	
18s-F1	CCTGGATACCGCAGCTAGGA	112 bp
18s-R1	GCGGCGCAATACGAATGCCCC	

### Western blot assay

USP48 and GAPDH protein levels were analyzed by western blotting [24]. Briefly, KNG cells were lysed, and 30 µg of protein was separated by 10% SDS-PAGE and transferred onto polyvinylidene fluoride membranes. Subsequently, all membranes were blocked with 5% skimmed milk diluted in TBS at 37˚C for 1 h and washed thrice with TBST. The membranes were then incubated with rabbit polyclonal USP48 (dilution 1:500, ab237765, Abcam, San Diego, CA, USA) and rabbit monoclonal antibodies against GAPDH (dilution 1:10,000, AF7021, Affinity) at 4˚C for 12 h. The membranes were washed thrice with TBST. Secondary antibodies were detected using Goat Anti-Rabbit IgG H&L (HRP) (dilution, 1:10,000, ab205718, Abcam) and visualized using an enhanced chemiluminescent reagent (PerkinElmer Life Sciences, MA, USA).

### Statistical analyses

The GEO database (https://www.ncbi.nlm.nih.gov/geo/) was used to analyze the differences in mRNA expression in the ovarian GCs of patients with PCOS. The search was conducted using the keywords [(granulosa cell) AND (polycystic ovary syndrome)] AND “Homo sapiens” in “GEO DataSets.” Further filtration was performed to identify datasets amenable to use “Analyze with GEO2R.” GSE106724, GSE137684, and GSE80432 were chosen to analyze differentially expressed genes in ovarian GCs of patients with PCOS according to GEO2R analysis. All data are presented as means ± standard deviation (SD) and were evaluated using SPSS analysis (SPSS Inc., U.S.A.). The significance of differences between control, H_2_O_2_, H_2_O_2_ + 0 µmol/L BAI, and H_2_O_2_ + 20 µmol/L BAI groups was assessed using one-way ANOVA with post hoc Dunnett’s multiple comparisons test used for comparison between two groups. Differences between the blank, siNC, and siUSP48 groups were assessed using one-way ANOVA. Statistical significance was set at *p* < 0.05.

## Results

### BAI treatment enhances the proliferation of H_2_O_2_-stimulated KGN cells

**Fig. 1:**
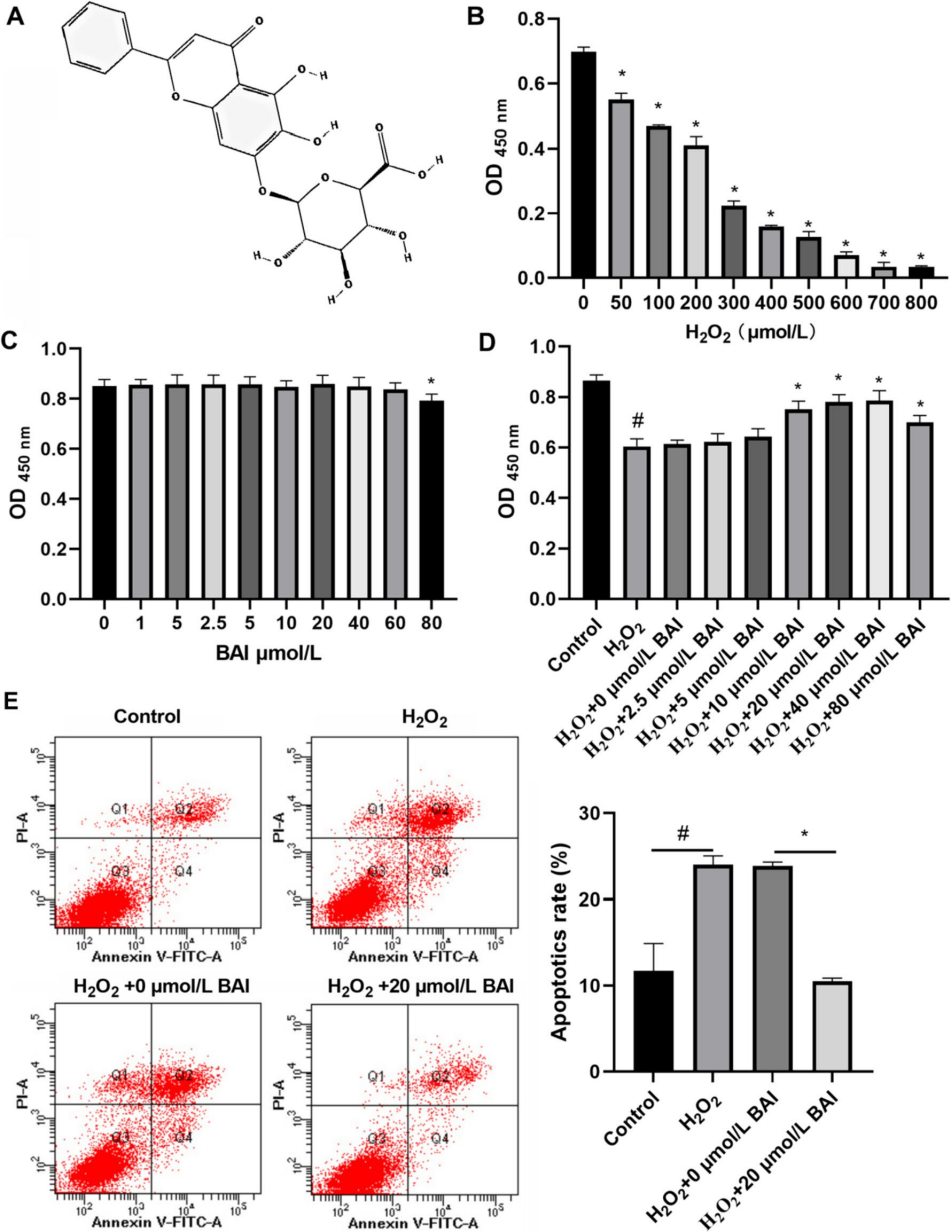
BAI promotes cellular proliferation and inhibits apoptosis of H_2_O_2_-stimulated KGN cells. **A** The molecular structure of BAI. **B** After H_2_O_2_ treatment for 24 h, the effect of different H_2_O_2_ concentrations on KGN proliferation was assessed using CCK8. *p < 0.05 vs. 0 µmol/L H_2_O_2_. **C** After BAI treatment at 24 h, the effect of different BAI concentrations on KGN proliferation was assessed using CCK8. *p < 0.05 vs. 0 µmol/L BAI. **D** After co-treatment with H_2_O_2_ and BAI for 24 h, the effects of different BAI concentrations on H_2_O_2_-stimulated KGN cell proliferation were analyzed using CCK8. ^#^*p* < 0.05 H_2_O_2_ vs. Control group; ^*^p < 0.05 vs. H_2_O_2_ group. **E** After co-treatment with H_2_O_2_ and BAI at 24 h, the effect of BAI on apoptosis of H_2_O_2_-stimulated KGN was analyzed using an apoptosis kit. *p < 0.05, ^#^*p* < 0.05

To identify the therapeutic potential of BAI against oxidative stress, KGN cells were treated with H_2_O_2_ for 24 h to mimic characteristics of oxidative stress and then treated with BAI. The molecular structure of BAI is shown in Fig. 1A. H_2_O_2_ inhibited KGN cell proliferation in a concentration-dependent manner (Fig. 1B). For BAI concentrations below 60 µmol/L, the treatment exhibited no notable impact on the proliferation of KGN cells when contrasted with the 0 µmol/L BAI treatment. However, 80 µmol/L BAI treatment could inhibit the proliferation of KGN cells (Fig. 1C). Next, 100 µmol/L H_2_O_2_ (≈IC_30_)-treated KGN cells were used as an in vitro model. At concentrations < 40 µmol/L, BAI promoted H_2_O_2_-induced KGN cell proliferation in a concentration-dependent manner (Fig. 1D). Proliferation in the 80 µmol/L treatment group was lower than that in the 40 µmol/L treatment group, indicating that BAI concentrations > 40 µmol/L inhibited proliferation (Fig. 1D). No significant difference in cell proliferation was observed between 20 and 40 µmol/L BAI treatment groups, whereas both the 20 and 40 µmol/L BAI treatment groups exhibited higher cell proliferation than the 10 µmol/L BAI treatment group (Fig. 1D). We selected the minimum concentration of BAI that demonstrated the most significant promotion of cell proliferation for subsequent experiments. Based on these results, we selected 20 µmol/L BAI for our subsequent studies. The findings also demonstrated that 100 µmol/L H_2_O_2_ promoted KGN cell apoptosis, whereas 20 µmol/L BAI treatment inhibited the apoptosis of H_2_O_2_-stimulated KGN cells (Fig. 1E).

### BAI ameliorated oxidative stress and inflammation in H_2_O_2_-stimulated KGN cells

**Fig. 2:**
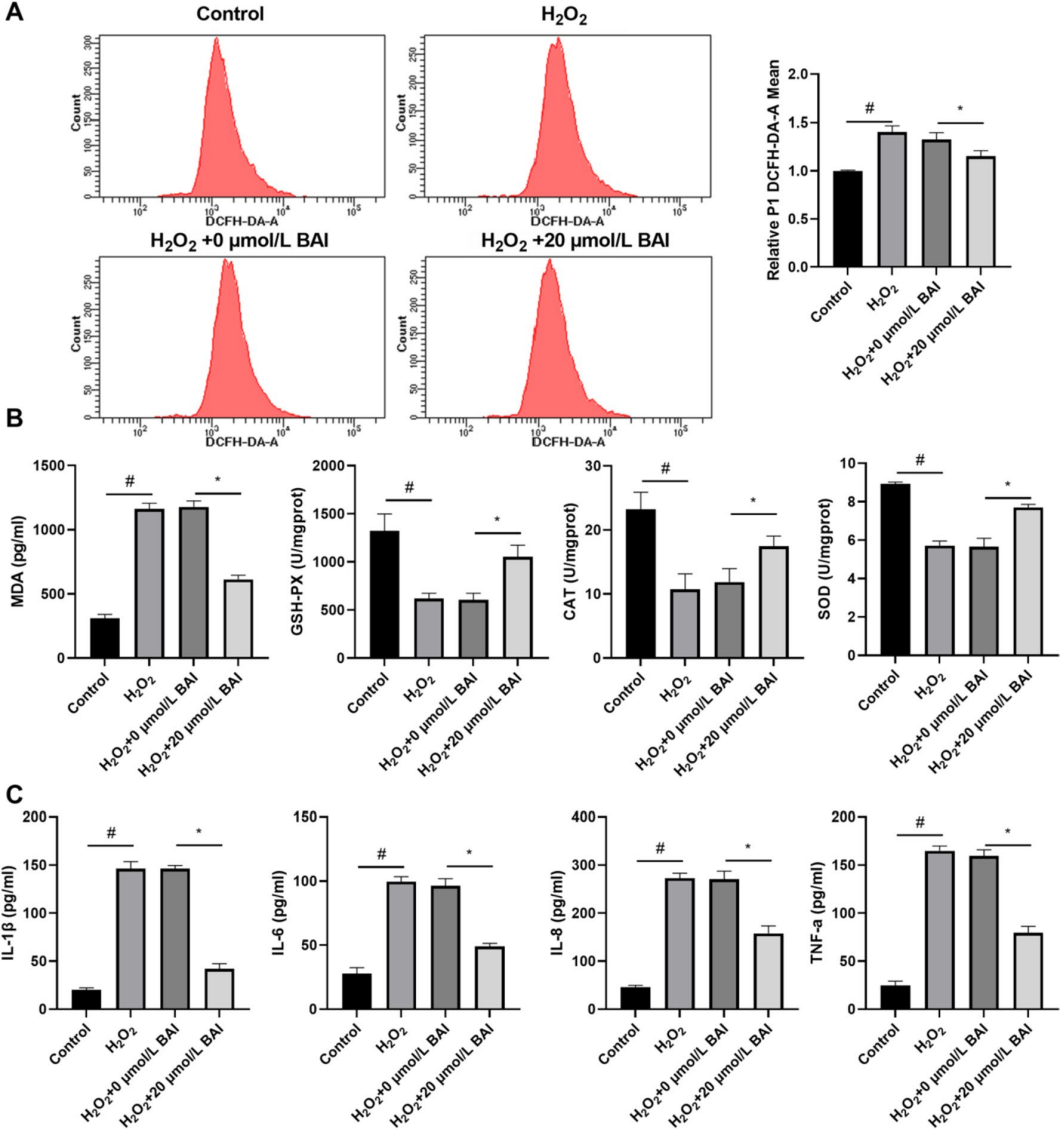
BAI treatment reduced oxidative stress and inflammation in H_2_O_2_-stimulated KGN cells. **A** ROS levels were measured by flow cytometry after BAI treatment for 24 h in H_2_O_2_-stimulated KGN cells. **B** MDA, GSH-PX, CAT, and SOD levels were measured using specific kits after BAI treatment for 24 h in H_2_O_2_-stimulated KGN cells. **C** IL-1β, IL-6, IL-8, and TNF-α levels were measured by ELISA after BAI treatment for 24 h in H_2_O_2_-stimulated KGN cells. ^#^*p* < 0.05 and *p < 0.05

To determine whether BAI mitigates oxidative stress and inflammation in H_2_O_2_-stimulated KGN cells, these cells were treated with 20 µmol/L BAI for 24 h. Compared to the control group, 100 µmol/L of H_2_O_2_ increased ROS and MDA levels while reducing GSH-PX, CAT, and SOD levels (Fig. 2A and B). Treatment with 20 µmol/L BAI reduced ROS and MDA levels while enhancing GSH-PX, CAT, and SOD levels in H_2_O_2_-stimulated KGN cells (Fig. 2A and B). Compared to the control group, 100 µmol/L H_2_O_2_ enhanced IL-1β, IL-6, IL-8, and TNF-α levels in KGN cells, whereas 20 µmol/L BAI reduced IL-1β, IL-6, and TNF-αlevels in H_2_O_2_-stimulated KGN cells (Fig. 2C).

### Screening for potential target genes of BAI in H_2_O_2_-stimulated KGN cells

**Fig. 3:**
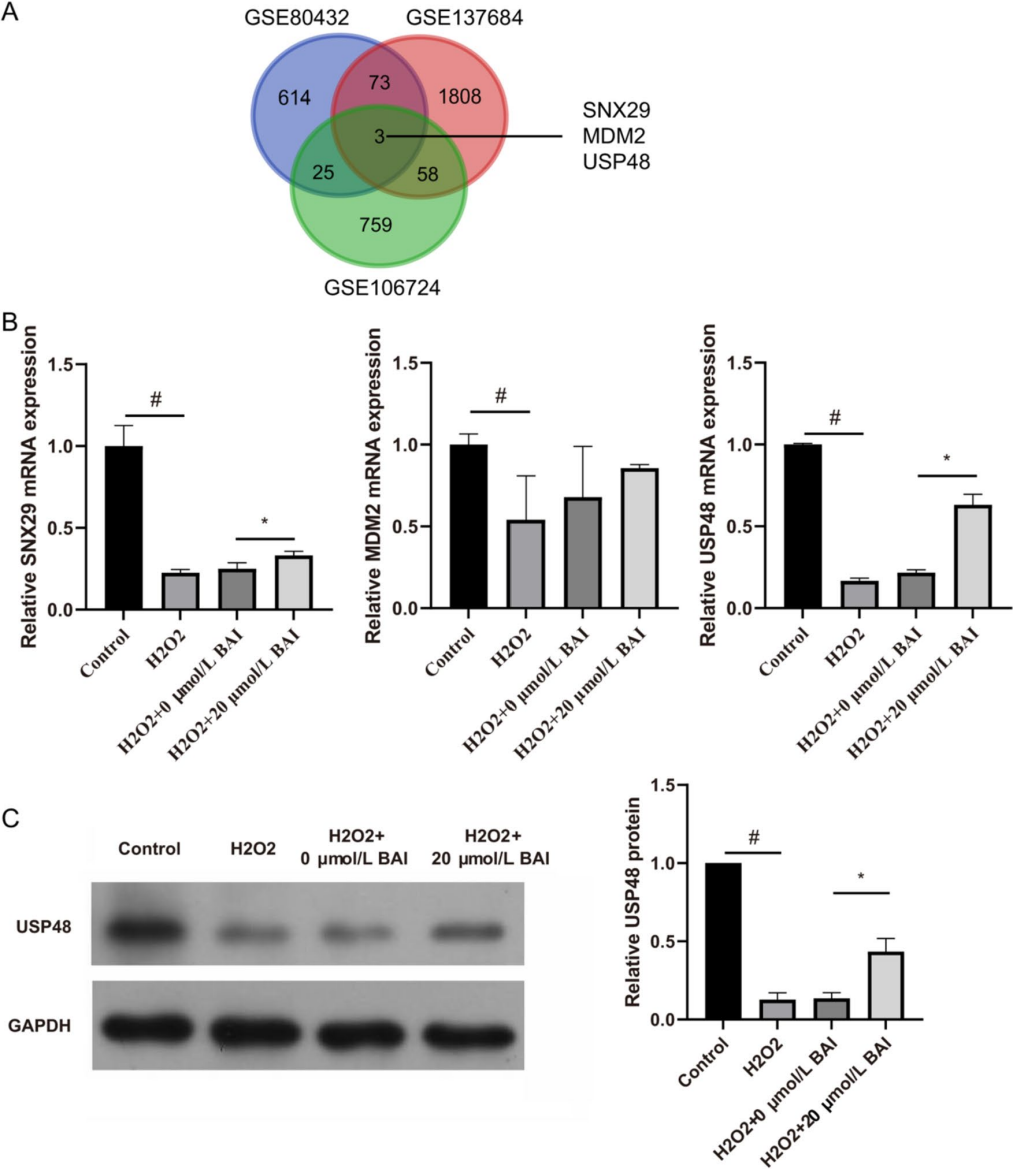
*USP48* is a potential target gene of BAI in H_2_O_2_-stimulated KGN cells. **A** The intersection of GSE106724, GSE137684, and GSE80432 included *SNX29, MDM2*, and *USP48* genes. **B** The mRNA levels of *SNX29, MDM2*, and *USP48* were measured by qRT-PCR after BAI treatment for 24 h in H_2_O_2_-stimulated KGN cells. **C** USP48 protein levels were measured by western blot after BAI treatment for 24 h in H_2_O_2_-stimulated KGN cells. ^#^*p* < 0.05 and *p < 0.05

According to GEO analysis, *SNX29, MDM2*, and *USP48* were found at the intersection of GSE106724, GSE137684, and GSE80432 (Fig. 3A). Compared with the control group, 100 µmol/L H_2_O_2_ enhanced the mRNA expression of *SNX29, MDM2*, and *USP48* in KGN cells. Furthermore, mRNA expression of only *USP48* was significantly enhanced after treatment with 20 µmol/L BAI for 24 h (Fig. 3B). Next, USP48 protein was significantly inhibited in H2O2-treated KGN cells. This inhibition was reversed significantly after treatment with 20 µmol/L BAI (Fig. 3C).

### USP48 knockdown reversed the enhancement effect of BAI on the proliferation of H_2_O_2_-induced KGN cells

**Fig. 4:**
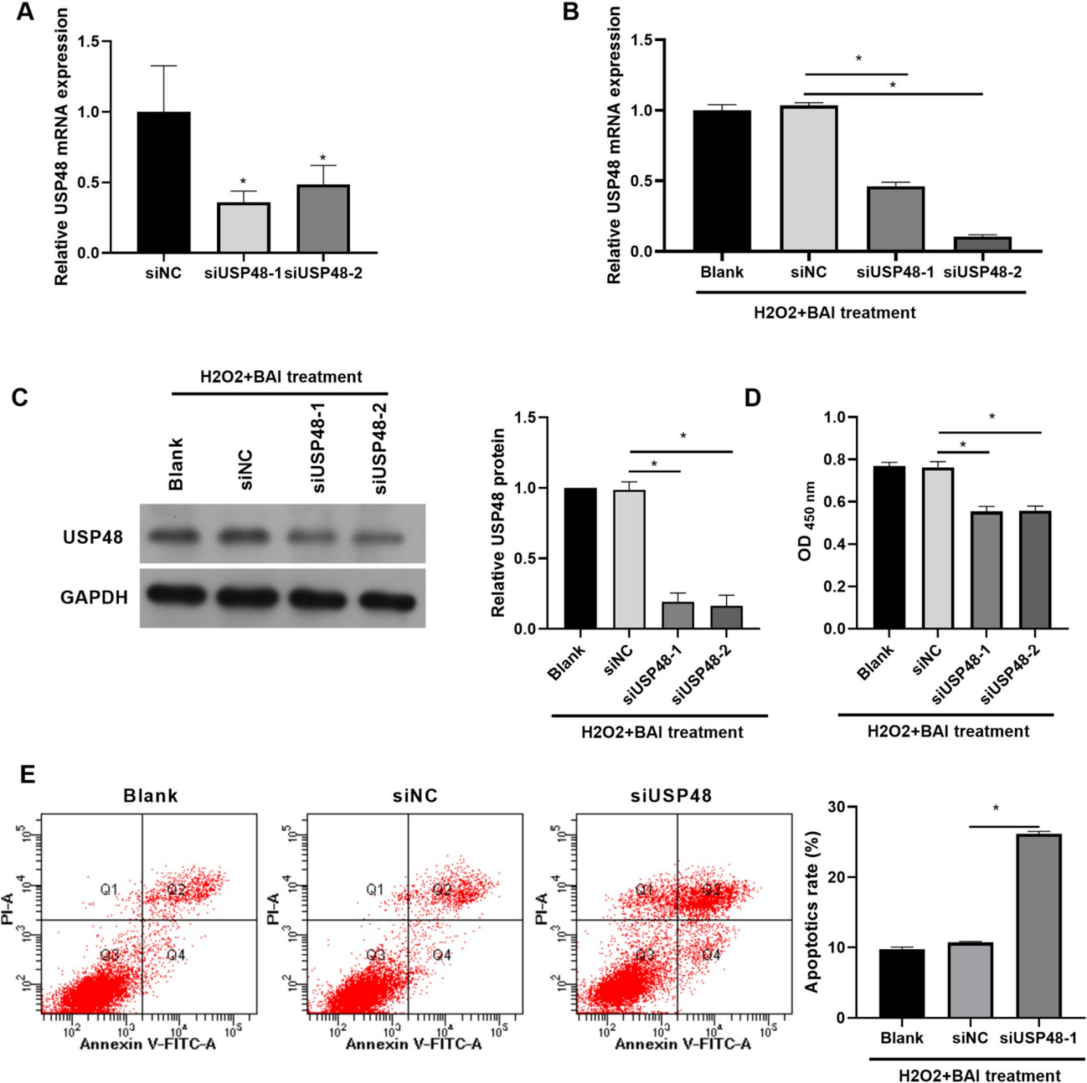
USP48 knockdown inhibited the proliferation of H_2_O_2_-stimulated and BAI-treated KGN cells. **A** USP48 mRNA levels in KGN cells were measured by qRT-PCR after siUSP48 transfection for 24 h without H_2_O_2_ and BAI treatment. **B** and **C** The mRNA and protein levels of USP48 in H_2_O_2_-stimulated KGN cells in response to BAI treatment were measured by qRT-PCR and western blotting after transfection with siUSP48-1 and siUSP48-2 at 24 h. **D** The effect of *USP48* knockdown on BAI-treated H_2_O_2_-induced KGN cell proliferation was analyzed using CCK8 after transfection at 24 h. **E** The effect of *USP48* knockdown on apoptosis in BAI-treated H_2_O_2_-stimulated KGN cells was analyzed using an apoptosis kit after siUSP48-1 transfection at 24 h. *p < 0.05

To examine whether USP48 influenced the inhibitory effect of BAI on the proliferation of H_2_O_2_-induced KGN cells, siUSP48 was transfected into KGN cells. USP48 mRNA expression was reduced in KGN cells after transfection (Fig. 4A). Then, the transfected KGN cells were treated with 100 µmol/L H_2_O_2_ and 20 µmol/L BAI for 24 h. The mRNA and protein levels of USP48 were reduced in transfected KGN cells (Fig. 4B and C). Compared to the H_2_O_2_ + BAI + siNC group, proliferation was inhibited in the H_2_O_2_ + BAI + siUSP48-1 and H_2_O_2_ + BAI + siUSP48-2 groups (Fig. 4D). In addition, apoptosis was enhanced in the H_2_O_2_ + BAI + siUSP48-1 group compared to that in the H_2_O_2_ + BAI + siNC group (Fig. 4E).

### USP48 knockdown reversed the inhibitory effects of BAI on oxidative stress and inflammation in H_2_O_2_-stimulated KGN cells

**Fig. 5:**
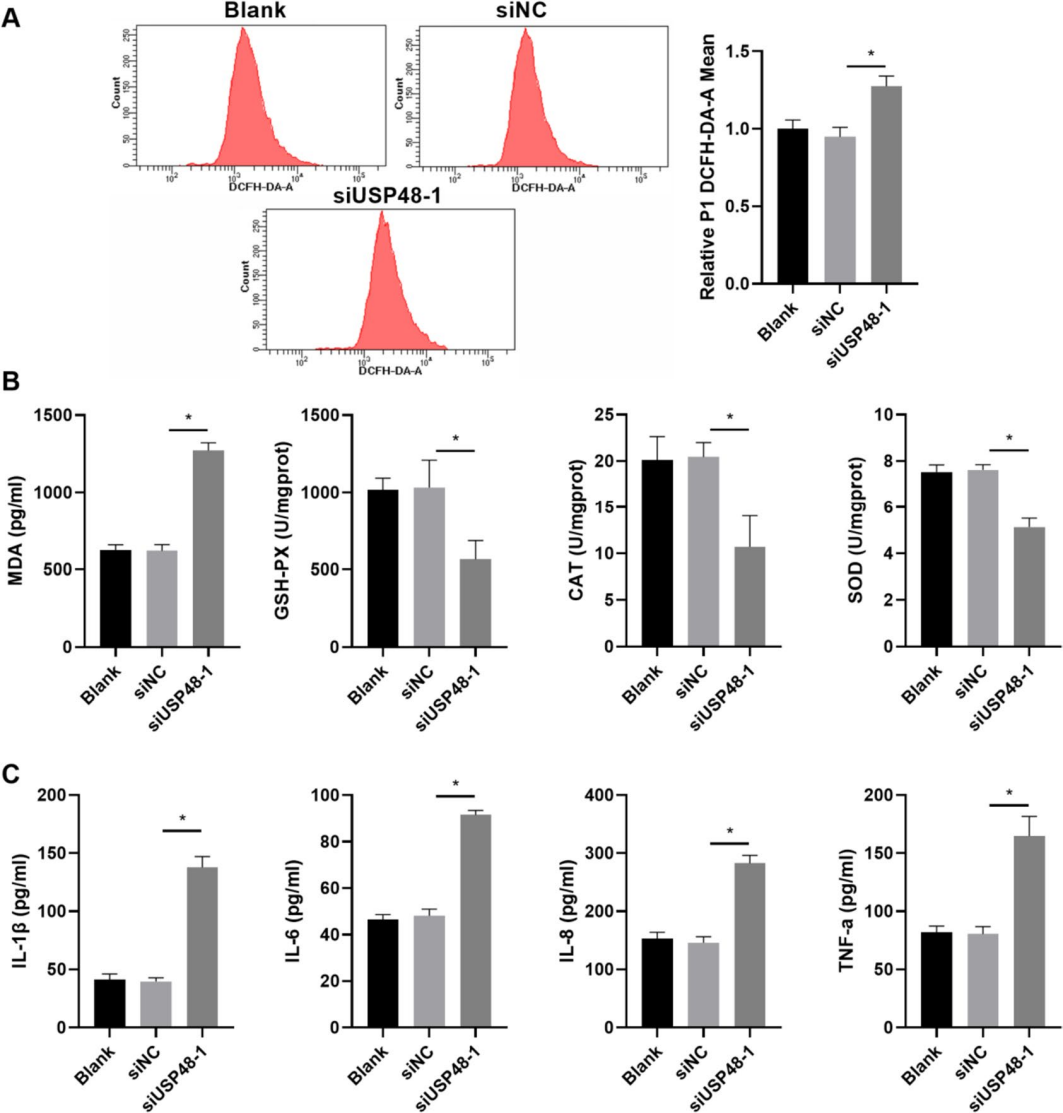
*USP48* knockdown reversed the inhibitory effect of BAI on oxidative stress and inflammation in H_2_O_2_-stimulated KGN cells. KGN cells in three groups were treated with 100 µmol/L H_2_O_2_ and 20 µmol/L BAI for 24 h. **A** ROS levels were measured by flow cytometry after siUSP48-1 transfection at 24 h. **B** The levels of MDA, GSH-PX, CAT, and SOD were measured after siUSP48-1 transfection at 24 h. **C** The levels of IL-1β, IL-6, IL-8, and TNF-α were measured by ELISA after siUSP48-1 transfection at 24 h. *p < 0.05

The transfected KGN cells were treated with 100 µmol/L H_2_O_2_ and 20 µmol/L BAI for 24 h. Compared with the H_2_O_2_ + BAI + siNC group, the levels of ROS and MDA were enhanced, whereas those of GSH-PX, CAT, and SOD were reduced in the H_2_O_2_ + BAI + siUSP48-1 group (Fig. 5A and B). The levels of IL-1β, IL-6, IL-8, and TNF-α were also increased in the H_2_O_2_ + BAI + siUSP48-1 group (Fig. 5C).

## Discussion

Oxidative stress involves a disturbance in the balance between prooxidants and antioxidants, which tends to favor an oxidized state. SOD, CAT, and GSH-Px are antioxidants that eliminate excess ROS and inhibit oxidative stress. Conversely, ROS, which are prooxidants, can generate free radicals and hydrogen peroxide when synthesized in excess, thereby promoting oxidative stress and induce cell apoptosis [25]. GCs are sensitive to oxidative stress, which contributes to impaired steroid synthesis and inflammation and consequently to the dysfunction and apoptosis of GCs [26, 27]. GCs support the energy metabolism of oocytes by providing substances that oocytes cannot produce, thereby supporting oocyte development and controlling meiotic division. Conversely, oocytes actively regulate the development and function of GCs by secreting various growth factors, promoting their proliferation and preventing apoptosis [28]. In addition, oocytes work in conjunction with estrogen to regulate the development and functionality of granulosa cells by promoting the expression of *FOXL2* [29]. Progesterone also inhibits apoptosis in granulosa cells [30]. Hence, while oocytes, estrogen, and progesterone regulate the proliferation of GCs, GCs support the normal development of oocytes and secretion of estrogen and progesterone. Therefore, oxidative stress-induced apoptosis of GCs can affect oocyte development. Conversely, abnormally developed oocytes further inhibit the proliferation of GCs. Abnormal oxidative stress and inflammation in GCs are closely associated with variations in chemical toxins (MC-LR), PM2.5, air pollution, and heavy metal (lead) [31–33]. Previous studies have indicated the activation of the PI3K-AKT, MAPK, FOXO, Nrf2, NF-κB, and AMPK pathways during the process of oxidative stress within GCs [7, 34]. H_2_O_2_ is an intermediate product of oxidative stress and is further degraded in water by CAT and GSH-PX. Elevated levels of H_2_O_2_ within the cell may lead to the generation of more toxic hydroxyl radicals, resulting in breaks and damage to the DNA strand, ultimately leading to apoptosis [25]. In the ovary, there is a transient increase in ROS levels after a surge in gonadotropins before ovulation; this elevation serves as a necessary signal for ovulation. It appears that primordial and small follicles are more resistant to H_2_O_2_ than other ovarian cell types, and H_2_O_2_ does not induce small follicular atresia in cultured neonatal ovaries nor does it result in apoptosis of primordial follicles [35]. However, H_2_O_2_ treatment enhances oxidative stress and inflammation, promoting GC apoptosis via the Nrf2/HO-1, ER stress, mTOR, and JNK/FOXO1 pathways [12, 17, 36, 37]. GC apoptosis is associated with a reduction in primordial follicular activity, PCOS, and other diseases associated with reduced ovarian functional reserves [28–30]. This study also showed that 100 µmol/L H_2_O_2_ treatment inhibited cell proliferation and promoted apoptosis, accompanied by increased MDA, IL-1β, IL-6, IL-8, and TNF-α levels and reduced SOD, CAT, and GSH-PX activities in KGN cells. These results suggest that H_2_O_2_ treatment enhanced oxidative stress and inflammation and promoted GC apoptosis.

BAI can inhibit cell skeleton rearrangement caused by loss of actomyosin stress fibers, regulate nuclear translocation of proteins, and suppress protein translation in ribosomes, thereby playing a beneficial role in disease treatment [38–40]. Accumulating evidence indicates that BAI reduces oxidative stress and inflammation. BAI treatment can reduce ROS levels, enhance cellular antioxidant capacity, and reduce intestinal epithelial cell apoptosis and skin aging under conditions involving H_2_O_2_ treatment [41, 42]. BAI treatment reduces oxidative stress and inflammation and ameliorates myocardial, nerve, and acute liver injuries [43–45]. In addition, 50 µM BAI treatment can reverse H_2_O_2_-induced GC cell apoptosis, upregulate the expression of P450arom and stAR, and increase the secretion of estradiol and progesterone [12]. Our results also showed that BAI exhibited antioxidant and anti-inflammatory effects in H_2_O_2_-stimulated KGN cells and reduced KGN apoptosis. Periplaneta americana, polydatin, and caffeic acid inhibit inflammation and oxidative stress, improve GC apoptosis, and enhance ovarian function [17, 32, 37]. The pharmacological mechanisms of these drugs are similar to those of BAI, in that they aim to improve oxidative stress and inflammation to exert their therapeutic effects. In addition, BAI treatment reduces testosterone concentrations and hormone secretion and promotes follicular development in PCOS models [13–15]. This evidence indirectly proves that BAI improves female reproductive system diseases, such as PCOS and premature ovarian failure. This may be related to improvements in oxidative stress-induced GC damage, further validating the therapeutic value of BAI. In addition, previous studies have indicated that BAI concentrations below 50 µM do not affect KGN survival, whereas concentrations above 80 µM can inhibit the survival of KGN [12, 15]. This study also demonstrates that under normal culture conditions, 80 µM BAI could inhibit KGN survival, whereas concentrations of BAI below 60 µM did not affect KGN survival. Under conditions involving H_2_O_2_ treatment, 10–40 µM BAI can improve KGN proliferation, with 20 µM and 40 µM being the most effective concentrations. Hence, the findings of this study propose that BAI concentrations ranging from 20 to 40 µM are advantageous for mitigating oxidative stress in KGN cells. Importantly, this concentration range aligns with the typical tolerable levels for KGN.

USP48 is a deubiquitination enzyme. Primarily localized in the nucleus, USP48 plays a crucial regulatory role in inherited retinal dystrophy (IRD) and ciliopathies by removing the ubiquitination of ARL3 and stabilizing UNC119a [22]. Similarly, USP48, primarily localized to the nucleus, impedes metabolic reprogramming to inhibit hepatocellular carcinoma development by removing K48-linked ubiquitination at the K33 and K128 sites of SIRT6 to stabilize SIRT6 [23]. USP48 promotes pyroptosis in cancer cells by removing K48-linked ubiquitination at positions K120 and K189 of the GSDME [21]. These studies indicate that USP48 can reverse disease progression via deubiquitination of targeted genes. In the present study, USP48 expression was reduced by H_2_O_2_ treatment and restored by BAI treatment. USP48 knockdown promoted inflammation, oxidative stress, and apoptosis in BAI-treated H_2_O_2_-stimulated KGN cells, indicating that USP48 knockdown abrogated the protective effects of BAI. These results show that BAI inhibited inflammation and oxidative stress by enhancing USP48 protein expression. In addition, these results showed that the functionality of USP48 is associated with inflammation and oxidative stress in GCs. Notably, USP48 inhibits NF-κB activity by regulating RelA deubiquitination to reduce cell inflammation and apoptosis [46, 47]. The activation of the PI3K/AKT/NF-κB signaling pathway in GCs of patients with PCOS contributes to an increase in the levels of ROS, IL-1β, IL-6, IL-8, and TNF-α [48]. Exposure of GCs to lead inhibits the expression of NRF2, activates NF-κB, increases levels of ROS and TNF-a, and decreases the expression of the antioxidant factors SOD and CAT [31]. Hence, NF-κB signaling pathway can enhance inflammation and oxidative stress in GCs. It may act as a key signaling pathway that links USP48 to inflammation and oxidative stress. However, the regulatory role of USP48 in the NF-κB pathway requires further research.

This study has some limitations. Firstly, the expression of USP48 in the ovarian tissue of patients with PCOS is not yet fully understood, precluding a definitive determination of its precise role in the pathogenesis of PCOS. Second, it remains unclear whether BAI directly binds to USP48 or regulates USP48 through other pathways, necessitating further research to elucidate their regulatory mechanisms. Additionally, the target genes and signaling pathways regulated by USP48, such as NF-KB, require further investigation to reveal their detailed mechanisms.

## Conclusion

In summary, 20 µmol/L BAI treatment improved cell proliferation and reduced oxidative stress and inflammation by enhancing USP48 protein expression in 100 µmol/L H_2_O_2_-stimulated KGN cells. Thus, BAI could be used as a clinical drug to ameliorate oxidative stress-induced damage in GCs.

### Supplementary Information

**Additional file 1.**

## Data Availability

The datasets used and/or analyzed in the current study are available from the corresponding author upon reasonable request.

## Abbreviations

BAI: Baicalin
CAT: Catalase
CCK-8: Cell Counting Kit-8
GCs: Granulosa cells
GSH-Px: Glutathione peroxidase
IL-1β: Interleukin 1β
MDA: Malondialdehyde
PCOS: Polycystic ovarian syndrome
ROS: Reactive oxygen species
SOD: Superoxide dismutase
TNF-α: Tumor necrosis factor-α
