# Supplementary material for: Baicalin enhances proliferation and reduces inflammatory-oxidative stress effect in H2O2-induced granulosa cells apoptosis via USP48 protein regulation
Source: BMC Complement Med Ther. 2024 Jan 20;24:42. doi: 10.1186/s12906-024-04346-z (PMC10799411; doi:10.1186/s12906-024-04346-z)
Supplement: Supplementary file 1 — Additional file 1. [file 12906_2024_4346_MOESM1_ESM.pdf]

Figure 3C

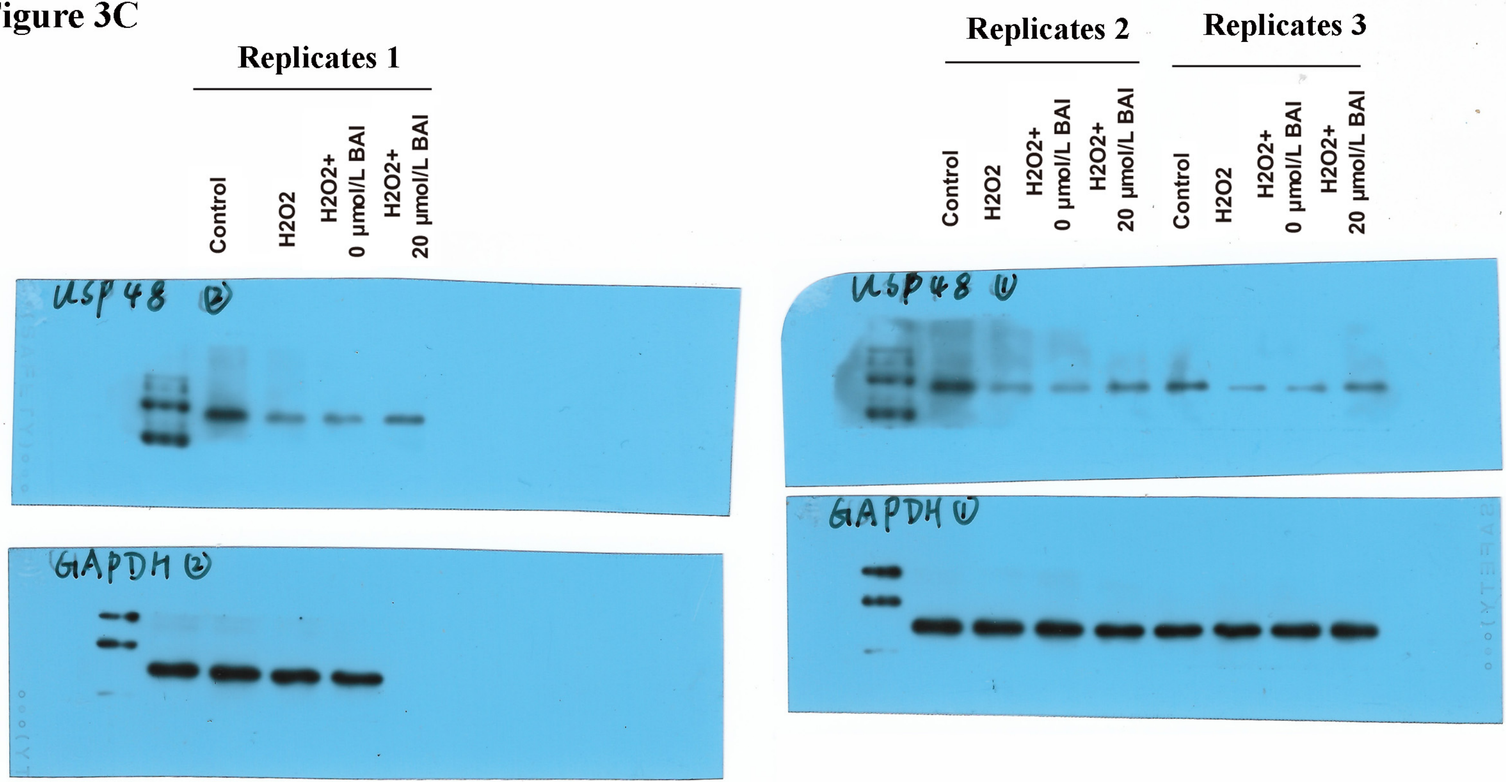

Replicates 1
Control
H2O2
H2O2+ 0 μmol/L BAI
H2O2+ 20 μmol/L BAI
Replicates 2
Replicates 3
Control
H2O2
H2O2+ 0 μmol/L BAI
H2O2+ 20 μmol/L BAI
Control
H2O2
H2O2+ 0 μmol/L BAI
H2O2+ 20 μmol/L BAI
USP48 ②
GAPDH ②
USP48 ①
GAPDH ①

Figure 4C

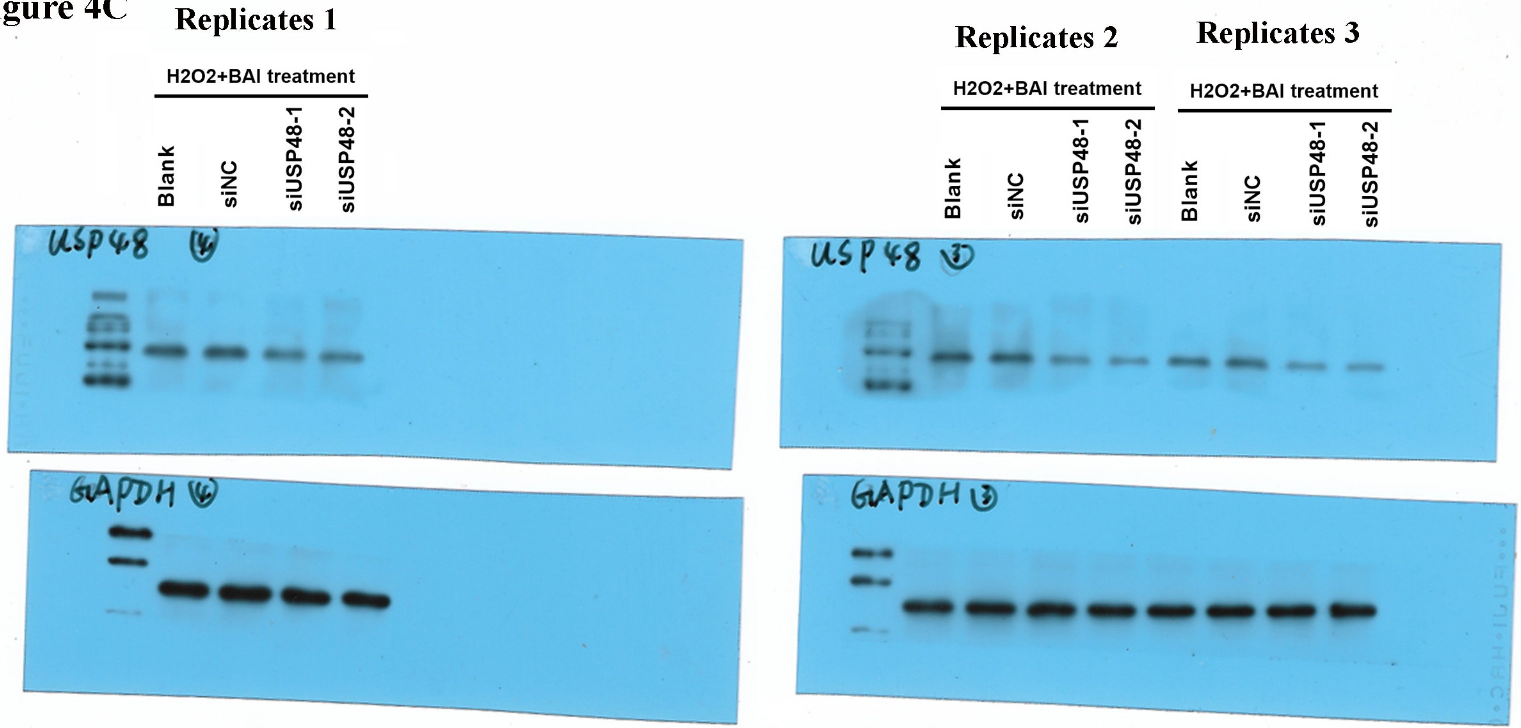

Replicates 1
H2O2+BAI treatment
Blank
siNC
siUSP48-1
siUSP48-2
USP48
GAPDH
Replicates 2
H2O2+BAI treatment
Blank
siNC
siUSP48-1
siUSP48-2
Replicates 3
H2O2+BAI treatment
Blank
siNC
siUSP48-1
siUSP48-2
USP48
GAPDH
